# Optimal strategies and cost-benefit analysis of the ${\varvec{n}}$-player weightlifting game

**DOI:** 10.1038/s41598-022-12394-z

**Published:** 2022-05-19

**Authors:** Diane Carmeliza N. Cuaresma, Erika Chiba, Jerrold M. Tubay, Jomar F. Rabajante, Maica Krizna A. Gavina, Jin Yoshimura, Hiromu Ito, Takuya Okabe, Satoru Morita

**Affiliations:** 1 Graduate School of Science and Technology, Shizuoka University, Hamamatsu, Shizuoka 423-8561 Japan; 2 Institute of Mathematical Sciences and Physics, College of Arts and Sciences, University of the Philippines Los Baños, 4031 Laguna, Philippines; 3 Graduate School of Informatics, Nagoya University, Furo-cho, Chikusa-ku, Nagoya, 464-8601 Japan; 4 Department of International Health and Medical Anthropology, Institute of Tropical Medicine, Nagasaki University, Nagasaki, 852-8523 Japan; 5 Department of Biological Sciences, Tokyo Metropolitan University, Hachioji, Tokyo, 192-0397 Japan; 6 The University Museum, University of Tokyo, Bunkyo-ku, Tokyo, 113-0033 Japan; 7 Graduate School of Integrated Science and Technology, Shizuoka University, Hamamatsu, Shizuoka 423-8561 Japan

**Keywords:** Evolution, Evolutionary theory, Behavioural ecology, Theoretical ecology, Animal behaviour

## Abstract

The study of cooperation has been extensively studied in game theory. Especially, two-player two-strategy games have been categorized according to their equilibrium strategies and fully analysed. Recently, a grand unified game covering all types of two-player two-strategy games, i.e., the weightlifting game, was proposed. In the present study, we extend this two-player weightlifting game into an $n$-player game. We investigate the conditions for pure strategy Nash equilibria and for Pareto optimal strategies, expressed in terms of the success probability and benefit-to-cost ratio of the weightlifting game. We also present a general characterization of $n$-player games in terms of the proposed game. In terms of a concrete example, we present diagrams showing how the game category varies depending on the benefit-to-cost ratio. As a general rule, cooperation becomes difficult to achieve as group size increases because the success probability of weightlifting saturates towards unity. The present study provides insights into achieving behavioural cooperation in a large group by means of a cost–benefit analysis.

## Introduction

Competition and cooperation in human or animal society are prevalent^1–5^. The existence and evolution of cooperation have been an interest in various disciplines^1,2,6–10^. Studies in game theory aim to develop criteria for selecting a strategy that maximizes gains and promotes cooperation^11–17^. Any situation can be considered a game if agents maximize their own gains by anticipating the actions of their opponents^18,19^. A game requires only a set of players, a set of strategies for each player, and corresponding pay-offs for each strategy in response to the strategies of other players. Rationality plays a strong role in determining what strategy a player should choose. Rational players maximize their expected gains without caring about societal goals^20–22^. Under the assumption of rationality, game theory finds an equilibrium of players' strategies at the point where no player can gain from changing his or her own strategy^20^. Game theory has received considerable attention from researchers as well as decision makers seeking to solve problems of conflict or cooperation^18^. Especially, the concept of the equilibrium strategy has been applied in behavioural science and psychology^2,8,23–25^, computer science^26,27^, economics and investments^6,28–30^, evolutionary biology^3,4,10^, and other fields.

Self-interest without regard to societal goals is best represented by the game known as the prisoner's dilemma (PD). In PD, two prisoners are to be convicted of a minor crime since prosecutors lack evidence to convict them of a major crime. Separated and with no way to communicate, the prisoners are offered a reduced sentence if they testify against each other. Rationality urges the two prisoners to betray one another, even though it is in their best interest to remain silent^2,31^. The stag hunt (SH) game also presents a social dilemma. In the SH, two hunters hunt for either a stag or a hare. They depend on each other in terms of which animal to hunt since they cannot kill the stag alone^8^. This results in two equilibria, one where both hunt a stag and another where both hunt a hare, but the best outcome is the former^8,32^. In the hawk-dove game (HD), which is equivalent to the chicken game (CH), the hawks are ready to fight for resources to drive the doves away, while the doves retreat whenever the hawks are around. This game has two equilibria of (Dove, Hawk) and (Hawk, Dove), while the highest pay-off is achieved for (Dove, Dove)^2^. Since cooperation and exploitation are prevalent in animals, game theory has been applied to study the evolution of animal behaviour, i.e., evolutionary game theory (EGT)^2,3^. In EGT, the assumptions of the rationality of players and the equilibrium of strategies in classical game theory are replaced with self-interest via Darwinian fitness and evolutionarily stable strategies (ESSs), respectively^3^. Strategies in EGT are behavioural phenotypes^3,4^.

The above three games and two trivial cases, C-dominant trivial (CT) and D-dominant trivial (DT), comprise the five classes of two-by-two games (two players, two strategies: cooperation and defection), represented by a $2\times 2$ matrix (Table 1)^33^. The pay-offs in two-by-two games are represented by four quantities, $R, T, S$, and $P$. The reward $R$ is received when the two players cooperate. The temptation $T$ is experienced by a player who then betrays the other player. The sucker $S$ is the experience of the betrayed player. The punishment $P$ is the pay-off when both players betray each other. Depending on the values of $R,T,S$ and $P$, two-by-two games are categorized into the aforementioned five types: prisoner's dilemma (PD; $T\ge R>P>S$), chicken game (CH; $T\ge R\ge S>P$), stag hunt game (SH; $R>T\ge P>S$), D-dominant trivial (DT; $T\ge P>R>S$) and C-dominant trivial (CT; $R>T\ge S>P$)^7,33–35^. DT and CT have equilibria of no dilemma, where all players defect or cooperate, respectively^7,34^. Recently, Yamamoto et al.^35^ introduced the two-person weightlifting game to unify all the five classes of dyadic games. In this game, each player either cooperates or defects in carrying a weight.

**Table 1:** Pay-off table of the two-person two-strategy game.

Row\Column	$C$	$D$
$C$	$\left(R,R\right)$	$\left(S,T\right)$
$D$	$\left(T,S\right)$	$(P,P)$

Studies on two-by-two games have contributed to understanding cooperation and dilemma in a social system. However, many societal concerns require cooperation and decisions of not just two individuals^5^. Multiple-player (or $n$-player) games have been studied extensively by researchers in various fields^12,26,36–40^, especially in behavioural science^41,42^ and other application areas^2,12,23,28,29,44,45^. The most studied $n$-player cooperative game is the public goods game (PGG)^32,41^, which is the $n$-player PD^32,33^. PGG models a society where members benefit equally from voluntary contributions (see refs.^33^ and^43^ for more discussion). Being an extension of PD, self-interest causes individuals to make non-cooperative decisions. The $n$-player CH is typically used to model social dilemmas caused by selfish individuals depleting a common resource^33^. Being equivalent to the $n$-player HD and snowdrift game, this game results in the coexistence of people who cooperate and people who free-ride on the work of others (see refs.^4,5^). The $n$-player SH still gives equilibria where all hunters cooperate to take down a stag or all defect to hunt hares instead (see ref.^8^). In these $n$-player games, it is generally expected that cooperation will diminish as the group size increases owing to the rational behaviour of self-interested individuals^4,20,38,41^.

The two-person two-strategy weightlifting game of Yamamoto et al*.*^35^ suggests a new way of investigating $n$-player games. In the present study, we extend this two-player game to an $n$-player game. Multiple-player games have now become possible to study in a unified manner. We investigate the conditions for pure strategy equilibria and optimal strategies, which we express with the success probability and the benefit-to-cost ratio of this model. Moreover, we provide the $n$-player extension of the classification conditions of two-by-two games according to the equilibrium and optimal strategies. In the final section, we discuss concrete examples of how the weightlifting game can explain behavioural cooperation in a large group.

## Model and results

### Preliminaries

To unify all the five classes of two-by-two games, Yamamoto et al.^35^ introduced the weightlifting game. In this game, each player either cooperates or defects in carrying a weight. Players who carry the weight pay a cost, $c\ge 0$. The weight is successfully lifted with probability ${p}_{i}$, where $i=\mathrm{0,1},2$ is the total number of cooperators and ${p}_{i}$ increases with the number of cooperators $i$. If the cooperators succeed, both players receive a benefit $b>0$. However, in case of failure, both players gain nothing. The pay-off of the cooperators is $b{p}_{i}-c$, and the pay-off of the defectors is $b{p}_{i}$ (Table 2). In terms of the parameters $\Delta {p}_{1}={p}_{1}-{p}_{0}$ and $\Delta {p}_{2}={p}_{2}-{p}_{1}$, which represents the increase in the probability of success due to an additional cooperator, the following inequalities are obtained for the pay-offs $R, T, S$, and $P$ (Table 1):

(i) $$\Delta {p}_{1}>c/b$$ for $$S>P$$,
(ii) $\Delta {p}_{2}>c/b$ for $R>T$, and
(iii) $$\Delta {p}_{1}+\Delta {p}_{2}>c/b$$ for $$R>P$$.

**Table 2:** Pay-off table of two-person weightlifting game.

Row\Column	$C$	$D$
$C$	$R:b{p}_{2}-c$	$S:b{p}_{1}-c$
$D$	$T:b{p}_{1}$	$P:b{p}_{0}$

PD satisfies only (iii), CH satisfies (i) and (iii), SH satisfies (ii) and (iii), DT satisfies none of the three conditions, and CT satisfies all three. In 2021, Chiba et al.^1^ studied the evolution of cooperation in society by incorporating environmental value in the weightlifting game. They found that the evolution of cooperation seems to follow a DT to DT trajectory, which can explain the rise and fall of human societies.

### The ${\varvec{n}}$-player weightlifting game

**Figure 1:**
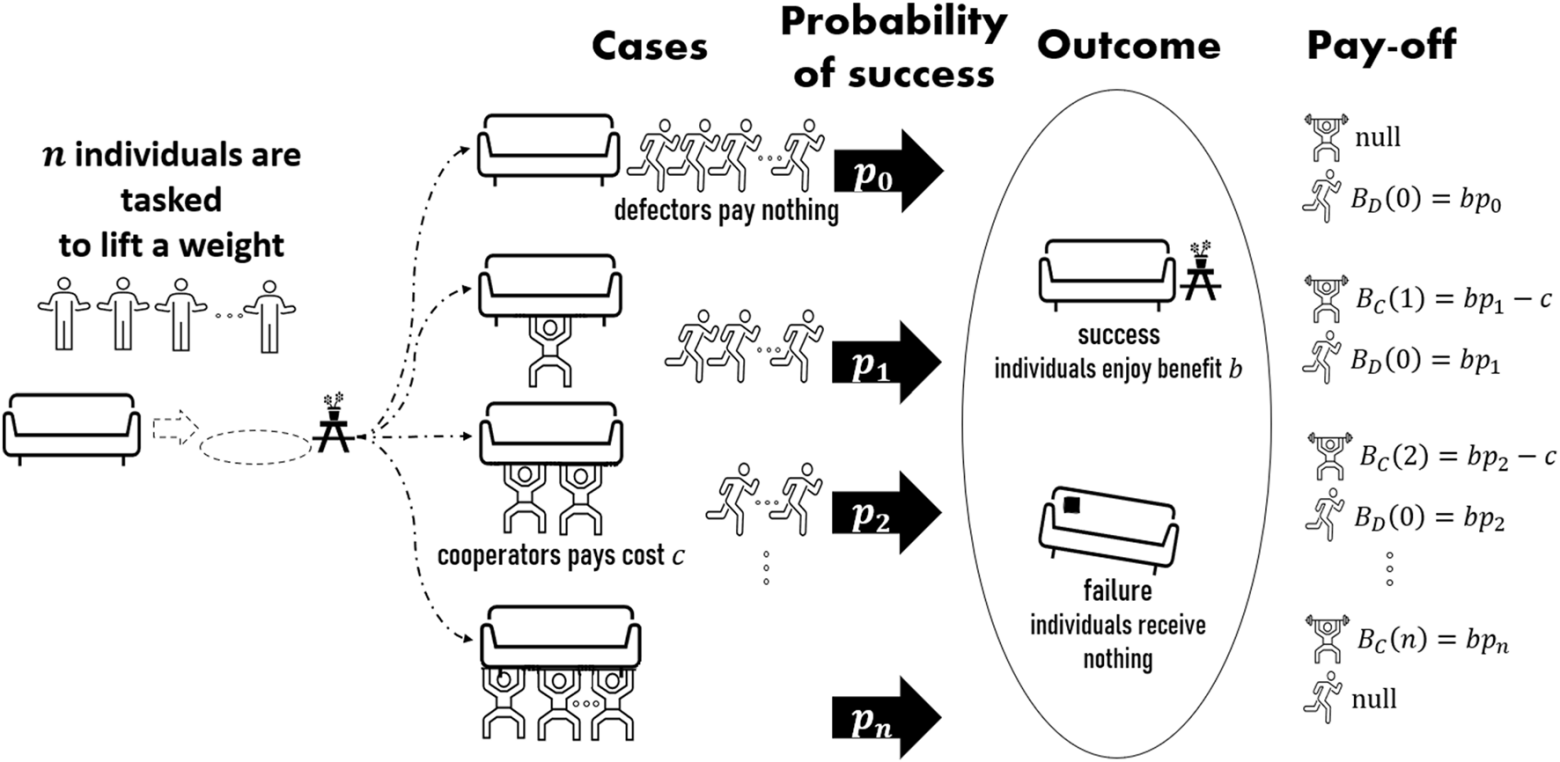
A schematic diagram of the *n*-player weightlifting game. In this game, players decide whether to cooperate or defect in carrying the weight. Cooperators need to pay a cost. The weightlifting can either succeed or fail. In case of success, all players receive a benefit. In case of failure, all players receive nothing. The player's pay-off depends on the benefit, cost and probability of success. Each player decides whether to cooperate or defect so as to maximize the expected gain.

In this study, we generalize the weightlifting game to $n$-players. Suppose $n$ self-interested and rational individuals selected from a population of infinite size. The $n$ players are asked to lift a weight. Each individual (or player) can decide to either carry the weight (cooperate, $C$) or not carry/pretend to carry the weight (defect, $D$). Players who decide to carry the weight can either succeed or fail. The probability of successful weightlifting is denoted by ${p}_{i}$, $i=\mathrm{0,1},\dots ,n$, where $i$ indicates the number of cooperators (henceforth, $i$ always represents the number of cooperators). The probability of success increases with the number of individuals cooperating, and it may remain less than unity even if all $n$ individuals cooperate. Players who decide to carry the weight pay a cost, $c\ge 0$, regardless of the outcome, while those who defect need not pay anything. If the cooperators succeed, all $n$ individuals receive a benefit $b\ge 0$. There is no penalty for failure. We use the expected gains/losses of the players as the pay-off. If there are $i-1$ cooperative players, then the pay-off of $j$ is ${B}_{C}\left(i\right)=b{p}_{i}-c$ when $j$ cooperates and ${B}_{D}\left(i-1\right)=b{p}_{i-1}$ when $j$ defects. The number of cooperators differs by one, since in ${B}_{C}\left(i\right)$, there is an additional cooperator, which is $j$ him- or herself. To decide whether to cooperate or defect, all players weigh their expected gain and rationally choose the option with the highest expected gain. The graphical outline of this game is illustrated in Fig. 1 (see also Supplementary Figure S1 for the flow of the game). The pay-off table for a four-player game is shown as an example in Table 3. Here, player $1$ is the innermost row (strategies are listed in the second column of the table), player $2$ is the innermost column (strategies are listed in the second row of the table), and the succeeding players take the succeeding rows or columns (we enter the first player as a row player and the following player as a column player and continue in this order). Each cell represents players' pay-offs, with the first component being the pay-off for the first player, the second for the second player, and so on. For instance, consider the entry in the first row and third column, where players $1, 2$ and $3$ cooperate but player $4$ defects. The pay-offs of players $1$ to $3$ are ${B}_{C}(3)$, while the pay-off of player $4$ is ${B}_{D}\left(3\right)$. In the above example, there are as many row players as column players because the number of players is even. However, we can have one more player in the rows than in the columns if there is an odd number of players.

**Table 3:** Pay-off table of four-player weightlifting game.

Row\Column		$C$		$D$	
		$C$	$D$	$C$	$D$
$C$	$C$	$\left({B}_{C}\left(4\right),{B}_{C}\left(4\right),{B}_{C}\left(4\right),{B}_{C}\left(4\right)\right)$	$\left({B}_{C}\left(3\right),{B}_{D}\left(3\right),{B}_{C}\left(3\right),{B}_{C}\left(3\right)\right)$	$\left({B}_{C}\left(3\right),{B}_{C}\left(3\right),{B}_{C}\left(3\right),{B}_{D}\left(3\right)\right)$	$\left({B}_{C}\left(2\right),{B}_{D}\left(2\right),{B}_{C}\left(2\right),{B}_{D}\left(2\right)\right)$
	$D$	$\left({B}_{D}\left(3\right),{B}_{C}\left(3\right),{B}_{C}\left(3\right),{B}_{C}\left(3\right)\right)$	$\left({B}_{D}\left(2\right),{B}_{D}\left(2\right),{B}_{C}\left(2\right),{B}_{C}\left(2\right)\right)$	$\left({B}_{D}\left(2\right),{B}_{C}\left(2\right),{B}_{C}\left(2\right),{B}_{D}\left(2\right)\right)$	$\left({B}_{D}\left(1\right),{B}_{D}\left(1\right),{B}_{C}\left(1\right),{B}_{D}\left(1\right)\right)$
$D$	$C$	$\left({B}_{C}\left(3\right),{B}_{C}\left(3\right),{B}_{D}\left(3\right),{B}_{C}\left(3\right)\right)$	$\left({B}_{C}\left(2\right),{B}_{D}\left(2\right),{B}_{D}\left(2\right),{B}_{C}\left(2\right)\right)$	$\left({B}_{C}\left(2\right),{B}_{C}\left(2\right),{B}_{D}\left(2\right),{B}_{D}\left(2\right)\right)$	$\left({B}_{C}\left(1\right),{B}_{D}\left(1\right),{B}_{D}\left(1\right),{B}_{D}\left(1\right)\right)$
	$D$	$\left({B}_{D}\left(2\right),{B}_{C}\left(2\right),{B}_{D}\left(2\right),{B}_{C}\left(2\right)\right)$	$\left({B}_{D}\left(1\right),{B}_{D}\left(1\right),{B}_{D}\left(1\right),{B}_{C}\left(1\right)\right)$	$\left({B}_{D}\left(1\right),{B}_{C}\left(1\right),{B}_{D}\left(1\right),{B}_{D}\left(1\right)\right)$	$\left({B}_{D}\left(0\right),{B}_{D}\left(0\right),{B}_{D}\left(0\right),{B}_{D}\left(0\right)\right)$

### Nash equilibrium and pareto optimal strategies

Here we present the Nash equilibrium and Pareto optimal strategies of the $n$-player weightlifting game in terms of the cost-to-benefit ratio $c/b$ and probability of success ${p}_{i}$. The Nash equilibrium consists of the best responses of each player. Players have no incentive to deviate from this strategy profile since deviation will not increase an individual’s pay-off if the other players maintain the same strategy. If ${B}_{C}(i)\ge {B}_{D}(i-1)$, the best response of player $j$ is to cooperate, but if ${B}_{C}(i)\le {B}_{D}(i-1)$, the best response is to defect.

We have $\Delta {p}_{i}={p}_{i}-{p}_{i-1}\ge 0$ for the increase in the probability of success because the probability ${p}_{i}$ increases with the number of cooperators $i$. It is convenient to divide cases depending on whether $\Delta {p}_{i}>c/b$ or $\Delta {p}_{i}<c/b$. We obtain the following results (see Supplementary Text for the derivations):

Result 1 *If *$\Delta {p}_{1}\le c/b$*, there is a Nash equilibrium at *$\left(D,D,\dots ,D\right)$*. The Nash equilibrium at *$(D,D,\dots ,D)$* is unique if and only if *$\Delta {p}_{i}<c/b$*, for all *$i=\mathrm{1,2},\dots ,n$*.*

Result 2 *If *$\Delta {p}_{n}\ge c/b$*, there is a Nash equilibrium at *$\left(C,C,\dots ,C\right)$*. The Nash equilibrium at *$(C,C,\dots ,C)$* is unique if and only if *$\Delta {p}_{i}>c/b$*, for all *$i=\mathrm{1,2},\dots ,n$*.*

Result 3 *There is a Nash equilibrium in the combination of strategies where* $i-1$ *players choose *$C$* and the rest of the players choose *$D$* if and only if *$\Delta {p}_{i}<c/b<\Delta {p}_{i-1}$*, for some *$i=\mathrm{2,3},\dots ,n$*.*

Result 1 shows that players have no incentive to cooperate when the cost relative to the benefit is (very) high, so much so that $\Delta {p}_{i}<c/b$, for all possible values of $i$. This case of all defection is a unique equilibrium, where no player can improve the pay-off by cooperating. In contrast, Result 2 shows that all players cooperate when the cost is sufficiently smaller than the benefit. Results 1 and 2 indicate that cooperation is determined by the relationship between the cost and the benefit; raising the benefit or lowering the cost can increase cooperation. There may be cases where full defection or cooperation is not a unique equilibrium (see cases 3 or 10, for example, in Table 4). The reason for this is covered by Result 3. This result shows the conditions for the existence of equilibria where only some individuals cooperate, which we will refer to as anti-coordination equilibria. Result 3 also implies the significance of an individual in promoting cooperation. For instance, when $\Delta {p}_{2}<c/b<\Delta {p}_{1}$, we have an equilibrium with a single cooperator. While there is a small chance of success, if an individual’s contribution to the probability of success is substantial, cooperation will exist. These three results cover all possible cases of pure equilibrium. The equilibria at $\left(D,D,\dots ,D\right)$ and at $\left(C,C,\dots ,C\right)$ are covered by Results 1 and 2, respectively, and the anti-coordination equilibria are covered by Result 3.

**Table 4:** Equilibrium strategies of a four-player weightlifting game.

Case	$\Delta {p}_{4}$	$\Delta {p}_{3}$	$\Delta {p}_{2}$	$\Delta {p}_{1}$	all-$D$	$1C,3D$	$2C,2D$	$3C,1D$	all-$C$
1	< $c/b$	< $c/b$	< $c/b$	< $c/b$	**X**				
2	< $c/b$	< $c/b$	< $c/b$	> $c/b$		**X**			
3	< $c/b$	< $c/b$	> $c/b$	< $c/b$	**X**		**X**		
4	< $c/b$	< $c/b$	> $c/b$	> $c/b$			**X**		
5	< $c/b$	> $c/b$	< $c/b$	< $c/b$	**X**			**X**	
6	< $c/b$	> $c/b$	< $c/b$	> $c/b$		**X**		**X**	
7	< $c/b$	> $c/b$	> $c/b$	< $c/b$	**X**			**X**	
8	< $c/b$	> $c/b$	> $c/b$	> $c/b$				**X**	
9	> $c/b$	< $c/b$	< $c/b$	< $c/b$	**X**				**X**
10	> $c/b$	< $c/b$	< $c/b$	> $c/b$		**X**			**X**
11	> $c/b$	< $c/b$	> $c/b$	< $c/b$	**X**		**X**		**X**
12	> $c/b$	< $c/b$	> $c/b$	> $c/b$			**X**		**X**
13	> $c/b$	> $c/b$	< $c/b$	< $c/b$	**X**				**X**
14	> $c/b$	> $c/b$	< $c/b$	> $c/b$		**X**			**X**
15	> $c/b$	> $c/b$	> $c/b$	< $c/b$	**X**				**X**
16	> $c/b$	> $c/b$	> $c/b$	> $c/b$					**X**

Result 4 *The number of equilibria of an *$n$*-player weightlifting game is at most* $\sum\nolimits_{i = 0}^{{\left\lfloor \frac{n}{2} \right\rfloor }} {C\left( {n,2i} \right)}$ *if *$n$* is even and* $\sum\nolimits_{i = 0}^{{\left\lfloor \frac{n}{2} \right\rfloor }} {C\left( {n,2i} \right) + 1}$ *if *$n$* is odd, where *$C\left( {n,2i} \right)$* denotes the combination of *$2i$* out of *$n$*.*

Result 4, on the other hand, gives the maximum number of equilibria in a weightlifting game. To illustrate this result, the equilibrium strategies (marked with **X**) of a four-player game are presented in Table 4. Notably, the one **X** in case 2 means not just one equilibrium but four equilibria: $(C,D,D,D)$, $\left(D,C,D,D\right),$ $\left(D,D,C,D\right)$ and $\left(D,D,D,C\right)$. The same applies to the other cases (except 1 and 16). As shown in Table 4, there can be at most three types of equilibrium (case 11): all-$D$, anti-coordination, and all-$C$. There is exactly one all-$D$ and exactly one all-$C$ strategy. However, there are $\left(2+2\right)!/(2!2!)=C(\mathrm{4,2})$ anti-coordination equilibria of two players cooperating and two players defecting; thus, there are at most eight equilibria in a four-player game. This finding is in accordance with Result 4: ${\sum }_{i=0}^{2}C(\mathrm{4,2}i)=C\left(\mathrm{4,0}\right)+C\left(\mathrm{4,2}\right)+C\left(\mathrm{4,4}\right)=8$.

In Pareto optimal strategies, players cannot increase their pay-offs by changing their strategy without also decreasing the other players' pay-offs. Owing to ${p}_{i}\le {p}_{i+1}$, ${B}_{D}\left(i\right)\le {B}_{D}\left(i+1\right)$ and ${B}_{C}\left(i\right)\le {B}_{C}(i+1)$. Thus, if a defector cooperates, the rest of the players will enjoy an increased pay-off. Moreover, some players will suffer from a decreased pay-off if cooperators decrease. In this case, we only have to check the condition that makes a strategy profile Pareto-dominated, i.e., when defectors cooperate.

Result 5 *Strategy* $\left(C,C,\dots ,C\right)$ *is Pareto optimal if and only if* ${\sum }_{j=1}^{n}\Delta {p}_{j}>c/b$.

Result 6 *The strategy profile with* $i$ *defectors, *$i=\mathrm{0,1},\dots ,n-1$*, is Pareto optimal if and only if *${\sum }_{j=i+1}^{n}\Delta {p}_{j}<c/b.$

In $(C,C,\dots ,C)$, the only way a player can deviate is to defect; thus, it is sufficient to check the condition where all-$D$ Pareto-dominates all-$C$. However, in the following result, which covers the remaining strategies, all-$D$ does not Pareto-dominate these strategies since defectors are disadvantaged. Furthermore, we know that ${\sum }_{j=1}^{n}\Delta {p}_{j}$ saturates towards unity. Thus, intuitively, cooperation is Pareto optimal unless $c$ is close to or greater than $b.$

### General properties of the ${\varvec{n}}$-player games

While Yamamoto et al*.*^35^ considered only the conditions that encourage cooperation, the violation of these conditions implicitly implies the satisfaction of the converse conditions. Thus, PD, SH and DT satisfying $\Delta {p}_{1}<c/b$ assures equilibrium at $(D,D)$. Moreover, PD and DT satisfying $\Delta {p}_{2}<c/b$ makes this equilibrium unique, according to Result 1. On the other hand, SH satisfying $\Delta {p}_{2}>c/b$ leads to another equilibrium at $(C,C)$ (Result 2). The anti-coordination equilibrium of CH is covered by the condition $\Delta {p}_{2}<c/b<\Delta {p}_{1}$ of Result 3. In addition, the condition $\Delta {p}_{1}+\Delta {p}_{2}>c/b$ (condition iii), which PD, CH, SH and CT satisfy, indicates that all-$C$ is more beneficial than all-$D$. As in Result 5, the counterpart of this condition for the $n$-player game is ${\sum }_{j=1}^{n}\Delta {p}_{j}>c/b$. Similarly, the inequality ${\sum }_{j=1}^{n}\Delta {p}_{j}<c/b$ indicates that all-$D$ is more beneficial than all-$C$ in Result 6 when $i=0$.

The five classes of two-by-two games are characterized by their equilibria and optimal strategies. All these games are unified under a single structure in the two-player weightlifting game. As an extension of the $n$-player game, the following correspondence occurs: With all-$C$ being the optimal strategy, PD has a unique equilibrium at all-$D$, CH has an anti-coordination equilibrium, SH has an equilibrium at both all-$C$ and all-$D$, CT has a unique equilibrium at all-$C$, and DT has a unique and optimal equilibrium at all-$D$. In the above results, we have shown the existence and uniqueness of an equilibrium and the existence of optimal strategies. In summary, we present the conditions and characterization of the $n$-player games in Table 5.

**Table 5:** Conditions for the $n$-player extension of two-by-two games.

	Conditions for equilibrium strategy	Conditions for optimal strategy	
PD	$\Delta {p}_{i}<c/b,\forall i\in P$	${\sum }_{j=1}^{n}\Delta {p}_{j}>c/b$	Results 1 and 5
CH	$\Delta {p}_{i}<c/b<\Delta {p}_{i-1},\exists i\in \left\{\mathrm{2,3},\dots ,n\right\}$	${\sum }_{j=1}^{n}\Delta {p}_{j}>c/b$	Results 3 and 5
SH	$\Delta {p}_{1}\le c/b$, $\Delta {p}_{n}\ge c/b$	${\sum }_{j=1}^{n}\Delta {p}_{j}>c/b$	Results 1, 2 and 5
CT	$\Delta {p}_{i}>c/b,\forall i\in P$	${\sum }_{j=1}^{n}\Delta {p}_{j}>c/b$	Results 2 and 5
DT	$\Delta {p}_{i}<c/b,\forall i\in P$	${\sum }_{j=1}^{n}\Delta {p}_{j}<c/b$	Results 1 and 6

### Illustration

**Figure 2:**
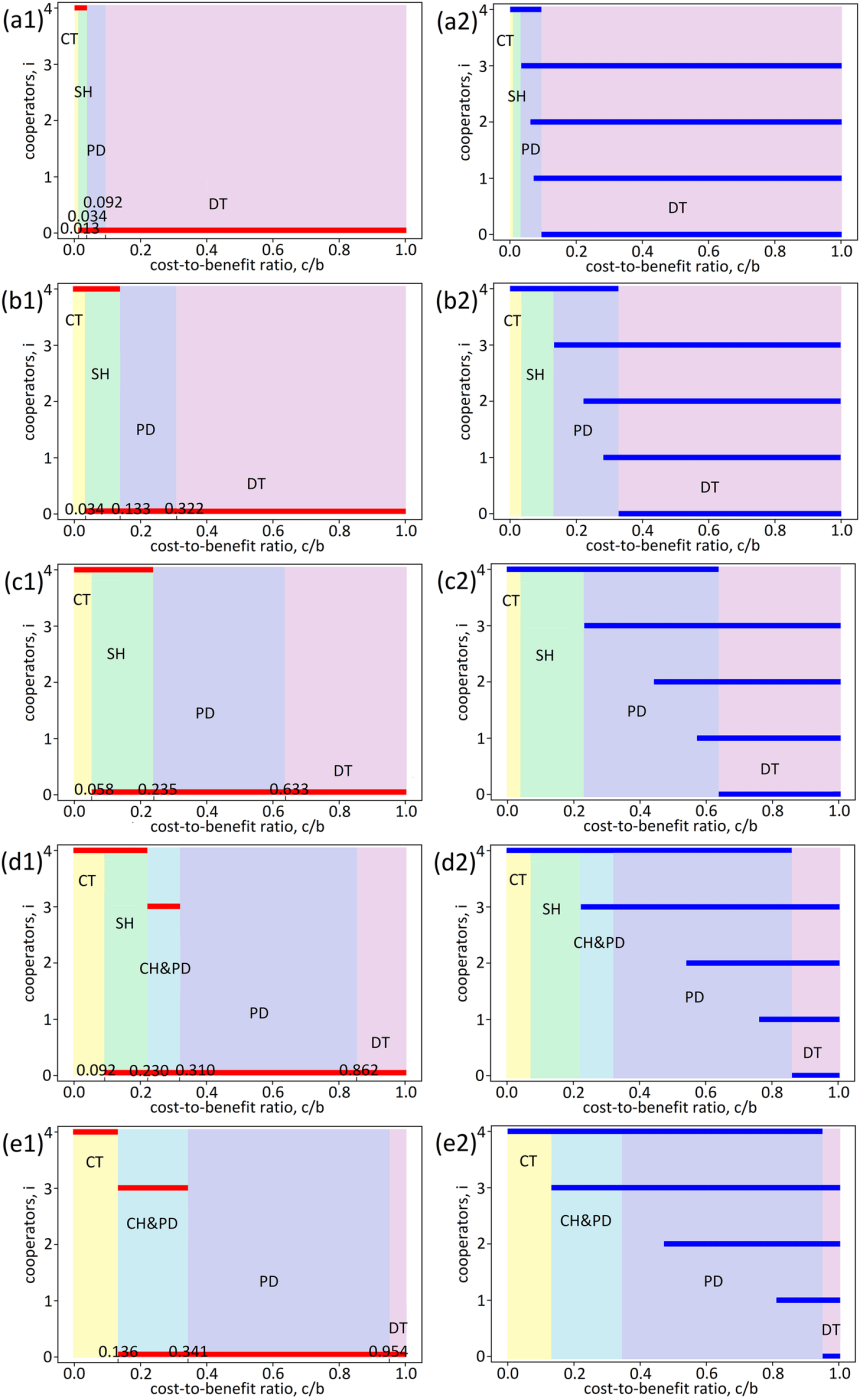
Equilibria and optimal strategies of the four-player weightlifting game. Nash equilibria (**a1**,**b1**,**c1**,**d1**,**e1**) and Pareto optimal strategies (**a2**,**b2**,**c2**,**d2**,**e2**). (**a1**,**a2**) $\mu =10$. (**b1**,**b2**) $\mu =20$. (**c1**,**c2**) $\mu =30$. (**d1**,**d2**) $\mu =40$. (**e1**,**e2**) $\mu =50$. The parameter regions for Nash equilibria and Pareto optimal strategies are as hatched in the *i*-$c/b$ plane, where *i* is the number of cooperators and $c/b$ is the cost-to-benefit ratio. We set $\sigma =50$ in all cases. All players cooperate for a small value of $c/b$ (CT), while they defect for a large value (DT).

**Figure 3:**
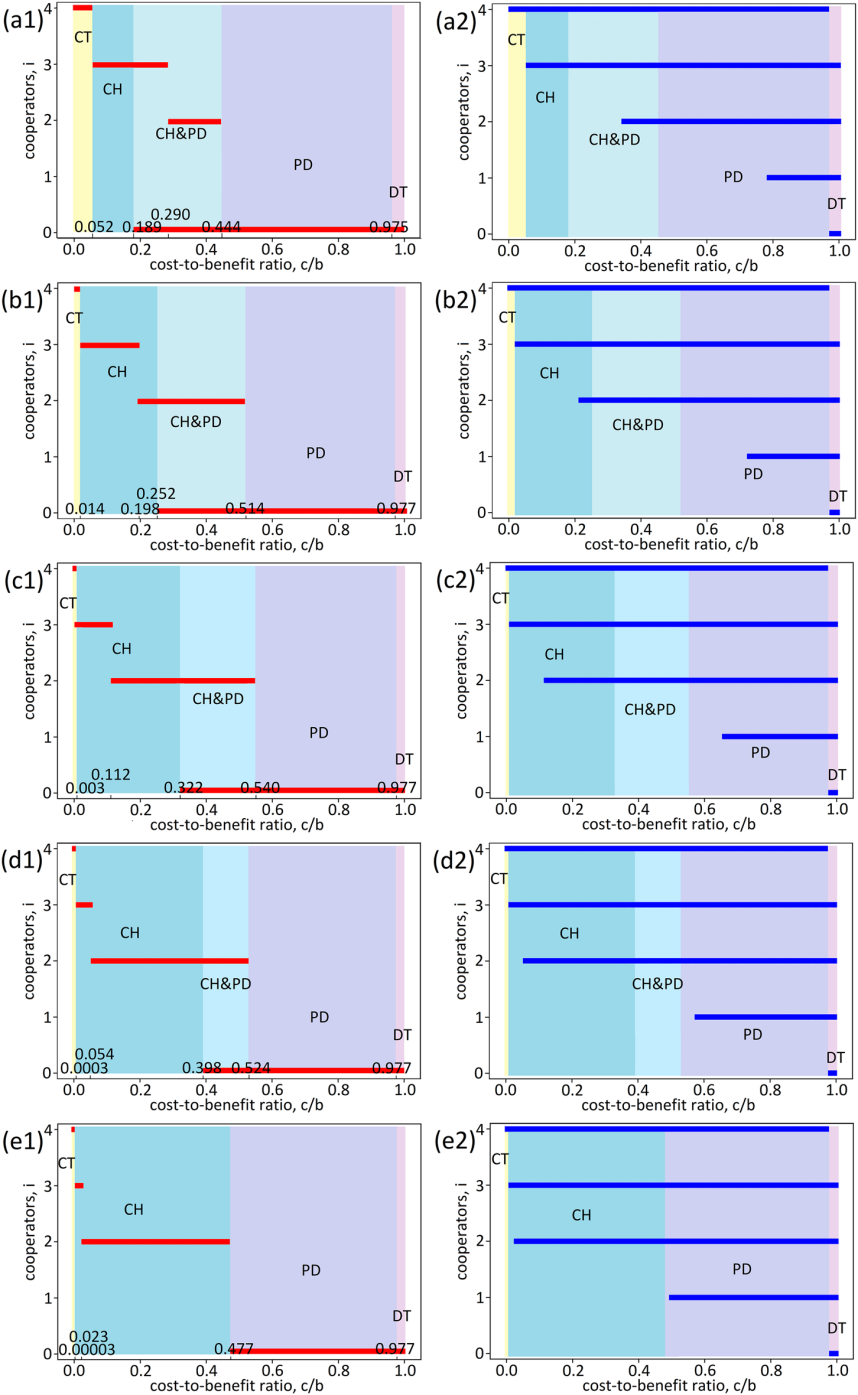
Equilibria and optimal strategies of the four-player weightlifting game. Nash equilibria (**a1**,**b1**,**c1**,**d1**,**e1**) and Pareto optimal strategies (**a2**,**b2**,**c2**,**d2**,**e2**). (**a1**,**a2**) $\mu =60$. (**b1**,**b2**) $\mu =70$. (**c1**, **c2**) $\mu =80$. (**d1**, **d2**) $\mu =90$. (**e1**,** e2**) $\mu =100$. See Fig. 2 and the text for details.

Let us consider a concrete example of lifting a weight of $W=100$ by four individuals (Figs. 2 and 3). The weight that each individual cay carry is normally distributed with mean $\mu$ and standard deviation $\sigma$. For $\mu =10$ and $\sigma =50$, we obtain $\Delta {p}_{1}=0.013,\Delta {p}_{2}=0.019,\Delta {p}_{3}=0.026$ and $\Delta {p}_{4}=0.034$ (Figs. 2a1, 2a2). The *n*-player CT obtains for $0<c/b<0.013$, SH for $0.013<c/b<0.034$, PD for $0.034<c/b<0.092$, and DT for $0.092<c/b<1$. In Figs. 2a1 and 2a2, we show the parameter regions for Nash equilibria and Pareto optimal strategies as hatched in the *i*-$c/b$ plane, where *i* is the number of cooperators and $c/b$ is the cost-to-benefit ratio. As $c/b$ increases, the number $i$ of cooperators drops from four to zero in Nash equilibria (Fig. 2a1). In Pareto optimal strategies, the number $i$ decreases from four to zero, while the range of $c/b$ for $i=0\sim 3$ reaches the right end point $c/b=1$ (Fig. 2a2). Note that the boundary values for the hatched bars are different for Nash equilibria (Fig. 2a1) and Pareto optimal strategies (Fig. 2a2). Similarly, we obtain Figs. 2b–e and 3a–e for $\mu$ from 20 to 100. The range for each game category varies depending on $\mu$. As $\mu$ increases, SH ceases to exist (Fig. 2e) while the coexistence CH&PD begins to appear (Fig. 2d) and disappear (Fig. 3e). A pure CH appears afterwards (Fig. 3b).

## Discussion

The present game is related to $n$-player Prisoner’s Dilemmas ($n$ PDs), or Public Goods games (PGG)^33,43^. Consider the public goods game played by $i$ cooperators and $j=n-i$ defectors. Each cooperator contributes $c$ to the public pool. Total contributions $ic$ is equally distributed among all players after multiplied by a factor $R$. Thus, each player gains $icR/n$. Since this quantity is compared with $b{p}_{i}$ of the weightlifting game, we see the correspondence of $cR$ to $b$ and $i/n$ to ${p}_{i}$. Accordingly, the success probability ${p}_{i}$ of the weightlifting game corresponds to the ratio of cooperators among all players in the public goods game. Unlike this specific case, however, in general, and in principle, the dependence of ${p}_{i}$ on the ratio $i/n$ can be nonlinear. The effect of this nonlinearity is properly taken into consideration in a general model of the weightlifting game. For instance, as a second example, let us consider $n$-player stag hunt dilemmas ($n$ SH). Pacheco et al.^32^ studied evolutionary dynamics in $n$ SH, where it is assumed that the “public goods” increases with the number of cooperators $i$ inasmuch as $i$ exceeds a certain threshold value $M$ while it is zero for $i<M$. This game is formally equivalent to replacing $R$ of PGG with $R\theta (i-M)$, where $\theta (x)$ is the Heaviside step function satisfying $\theta \left(x\right)=0$ for $x<0$ and $\theta \left(x\right)=1$ for $x\ge 0$. Consequently, $n$ SH is recovered by the weightlifting game under the assumption ${p}_{i}=i\theta (i-M)/n$, i.e., we need at least $M$ cooperators for the weightlifting to be successful (or to produce any benefits). A third example is provided by a $n$-player snowdrift game^46^, while it is more sophisticated. Souza et al.^46^ studied the $n$-person snowdrift game in which the cost paid by a cooperator depends on the number of cooperators, $i$, i.e., it is given by $C/\mathrm{max}(i,M)$, where $M$ is a minimum number of cooperators required for achieving a benefit and $\mathrm{max}\left(i,M\right)=i$ if $i\ge M$ and $M$ if $i<M$. Each player receives a benefit $B\theta (i-M)$, which is zero for $i<M$ and $B$ for $i\ge M$. This game is obtained by assuming ${p}_{i}=\theta \left(i-M\right)\times \mathrm{max}\left(i,M\right)/n=i\theta (i-M)/n$ as above, but with $b/c=nB/C$ depending on $n$ because each cooperator’s cost decreases with the total number. This explicit $n$-dependence of the benefit-to-cost ratio may reinforce the impact of group size, as mentioned just below.

It has been generally acknowledged that cooperation becomes difficult to achieve as group size increases ^4,20,37,41,46–48^. The effects of group size may be discussed from a static perspective^40^. For instance, the size dependence comes in through the decrease in the benefit-to-cost ratio as the number of players increases. When the total gain $W$ does not increase in proportion to the number of players $n$, the benefit of each player $b=W/n$ decreases as the number of players $n$ increases. Thus, the inequality $\Delta {p}_{i}<c/b$ should be met for all $i$ eventually, because the right-hand side increases in proportion to $n$. In other words, the larger the group, the less cooperative people will be. However, when the total gain $W$ increases in proportion to $n$, the right-hand side $c/b$ stays constant even if $n$ increases. In this case, the impact of group size on cooperation can be positive (or, to be precise, the negative effect of group size is mitigated when the benefits reaped by one individual do not reduce the benefits received by another). In fact, the impact in the latter case ($W\propto n$) has been studied as compared specifically against the former case ($W=$ const.) (see, e.g., refs.^49,50^ ). Recently, the emergence of cooperation in a large group has been extensively studied by means of dynamical models^32,48,50^. In this context, it should be remarked that the size effect may also come about as a result of dynamical, stochastic processes of how the numbers of players with different strategies vary, namely a genetic drift in evolutionary biology^47^. While assessing if the size effect due to genetic drift is positive or negative requires further assumptions than necessary for the present ‘static’ results, we made a calculation to find that the size effect, as evaluated from Eq. (2.5) of Kurokawa and Ihara^47^, is negative (Supplementary Text). Thus, we consider it an interesting future research direction to investigate population dynamics of the present game, especially to make a more specific comparison with these prior studies.

Several studies^8,32,46^ discuss a minimum number $M$ of players for cooperation, specifically anti-coordination, to exist. Our study can also supply this concept of threshold using the parameter $\Delta {p}_{i}$. A good example is provided by the concept of a ‘threshold’ in joining a strike, which is defined as the number of people in the strike for a given employee to join the strike (see ref.^51^). This number (threshold) may be different for a different individual. In fact, it is evaluated according to Result 3; an employee will join the strike under the condition $\Delta {p}_{i}<c/b<\Delta {p}_{i-1}$ when $i-2$ employees are in the strike. In the present model, the probability of success is used instead of the risk preference to evaluate the threshold value. When each individual has his/her own success probabilities $\Delta {p}_{i}$, the threshold can be different for each individual if the cost-to-benefit ratio $c/b$ is a fixed constant. Specifically, if $\Delta {p}_{2}<c/b<\Delta {p}_{1}$, a single employee (‘instigator’) will decide to start the strike, while it can be that the threshold becomes so high that the condition $\Delta {p}_{i}<c/b<\Delta {p}_{i-1}$ is not met for any $i$.

This ‘threshold’ behaviour is not unique to humans. Conradt and Roper^52^ studied social animals making communal decisions. The animals decide how long they conduct a communal activity, which is beneficial to the group but takes time away from their own personal activities^46,52^. Conradt and Roper^52^ named this loss of personal time the ‘synchronization cost’. They noted that the animals that stop communal activity earlier should have twice as much motivation as the others (‘double motivation’). If $i-1$ animals pursue communal activities, the animals to stop earlier (defect) are those that satisfy the condition $\Delta {p}_{i}<c/b<\Delta {p}_{i-1}$. In the present model, the motivation for communal activity is modelled with the probability of success.

In the previous section (Section “Illustration”), we presented that the game follows CT-SH-PD-DT, CT-SH-CH&PD–PD-DT, CT-CH&PD–PD-DT, CT-CH-CH&PD–PD-DT, and CT-CH-PD-DT (Figs. 2 and 3). This is consistent with the previous result^1^, while there are slight differences in the order and where SH appears. These trajectories may be used to explain the dynamical process of joining a strike^51^. We may regard $\mu$ as the employee’s rank in the company. The larger $\mu$, the higher the rank. When the consequences for joining the strike are minor, all employees are persuaded to join the strike (game type CT), regardless of the rank. As the consequences become severe, they are inclined not to join the strike, especially for those with a low rank $\mu$ (SH). If the consequences become more severe, those with a high rank $\mu$ are encouraged to leave the strike (PD). Not surprisingly, no employees will join the strike when faced with much more severe penalties (DT). This is just an example. Many other cases can be analysed from the perspective of the present study.

The weightlifting game does not only pertain to the physical act of carrying a load, as we have seen in the examples provided above. The probability of successful weightlifting ${p}_{i}$ can also be interpreted in different ways. For example, it can be interpreted as the probability of not depleting the public resource, the probability of driving the other species away, or the probability of taking down the stag. We can also regard ${p}_{i}$ as the probability of $i$ individuals to reproduce and for its species to prevent extinction. Many other interpretations are possible. This study can be extended in several ways: (1) by incorporating environmental effects, such as spatial and temporal parameters; (2) by generalizing the cost $c$ and benefit $b$ to depend on the players; (3) by considering the risk aversion of the players; and (4) by considering pre-play communication. The evolution from one class of game to another is also worth studying.

## Supplementary Information

Supplementary Information.

## Data Availability

This study is theoretical and does not use any data. In the illustration, all results can be computed directly from the values and formulas presented in the text.
