# Supplementary material for: Optimal strategies and cost-benefit analysis of the n-player weightlifting game
Source: Sci Rep. 2022 May 19;12:8482. doi: 10.1038/s41598-022-12394-z (PMC9120137; doi:10.1038/s41598-022-12394-z)
Supplement: Supplementary file 1 — Supplementary Information. [file 41598_2022_12394_MOESM1_ESM.pdf]

Supplementary Information: Optimal strategies and cost-benefit analysis of the *n*-player weightlifting game

Diane Carmeliza N. Cuaresma, Erika Chiba, Jerrold M. Tubay, Jomar F. Rabajante, Maica Krizna A. Gavina, Jin Yoshimura, Hiromu Ito, Takuya Okabe and Satoru Morita

Supplementary Text

1. Derivation of the results

Here we provide detailed derivations of the results in the main text.

> Result 1 *If $\Delta p_1 \leq c/b$, there is a Nash equilibrium at $(D, D, \ldots, D)$. The Nash equilibrium at $(D, D, \ldots, D)$ is unique if and only if $\Delta p_i < c/b$, for $i = 1,2, \ldots, n$.*

If $\Delta p_1 \leq c/b$ or $B_D(0) \geq B_C(1)$, then any player has no incentive to cooperate, i.e., $(D, D, \ldots, D)$ is a Nash equilibrium. From among i cooperators, an arbitrary player j will defect if $B_D(i-1) > B_C(i)$, which is equivalent to $bp_{i-1} > bp_i - c$, i.e., $\Delta p_i < c/b$. If this inequality holds for $i = 1,2, \ldots, n$, there can be no Nash equilibrium except $(D, D, \ldots, D)$.

> Result 2 *If $\Delta p_n \geq c/b$, there is a Nash equilibrium at $(C, C, \ldots, C)$. The Nash equilibrium at $(C, C, \ldots, C)$ is unique if and only if $\Delta p_i > c/b$ for $i = 1,2, \ldots, n$.*

Result 2 can be derived in the same manner as Result 1. If $\Delta p_n \geq c/b$, all players will cooperate since $B_C(n) > B_D(n-1)$, and a Nash equilibrium will exist at $(C, C, \ldots, C)$. If an arbitrary player j cooperates regardless of the number of cooperators $i-1$, then $B_C(i) > B_D(i-1)$ or $\Delta p_i > c/b$. No other Nash equilibrium exists except for $(C, C, \ldots, C)$ if the inequality holds for all possible values of i.

> Result 3 *There is a Nash equilibrium in the combination of strategies where $i - 1$ players choose C and the rest of the players choose D if and only if $\Delta p_i < c/b < \Delta p_{i-1}$, for some $i = 2,3, \ldots, n$.*

Suppose a strategy with $i-1$ cooperators is a Nash equilibrium. No cooperator has an incentive to defect, so $B_C(i-1) > B_D(i-2)$ or $\Delta p_{i-1} > c/b$. A defector, on the other hand, has no incentive to change strategy, so $B_D(i-1) > B_C(i)$ or $\Delta p_i < c/b$. Keeping in mind that a strategy profile can only contain pay-offs that are all $B_D(0)$, all $B_C(n)$, or combinations of $B_D(i)$ and $B_C(i)$ for $i = 1,2, \ldots, n-1$, the converse should follow.

Result 4 *The number of equilibria of an* n*-player weightlifting game is at most* $\sum_{i=0}^{\left\lfloor\frac{n}{2}\right\rfloor} C(n,2i)$ *if* n *is even and* $\sum_{i=0}^{\left\lfloor\frac{n}{2}\right\rfloor} C(n,2i)+1$ *if* n *is odd, where* $C(n,2i)$ *denotes the combination of* $2i$ *out of* n*.*

Given n players, there are $C(n,i)$ combinations of i cooperators from n players. If $\Delta p_1 < c/b$, there is an equilibrium at all-D by Result 1, i.e., 0 cooperators, so that we have $C(n,0)=1$ equilibrium. If $\Delta p_n > c/b$, there is an equilibrium at all-C by Result 2, i.e., n cooperators, so that we have $C(n,n)=1$ equilibrium. For the anti-coordination equilibrium, we need to consider how $\Delta p_2, \dots, \Delta p_{n-1}$ behaves. The condition $\Delta p_i < c/b < \Delta p_{i-1}$ of Result 3 is vital in counting the remaining equilibria. Since all-D is obtained if $\Delta p_1 < c/b$, the pairing for Δp_i and Δp_{i-1} should start with $i=3$, i.e., Δp_3 is paired with Δp_2, Δp_5 is paired with Δp_4, and so on. Consequently, the number of cooperators must be even-numbered. If n is even, there are $C(n,2)+C(n,4)+\cdots+C(n,n-2)$ anti-coordination equilibria. If n is odd, there are $C(n,2)+C(n,4)+\cdots+C(n,n-3)$ anti-coordination equilibria.

Result 5 *Strategy* $(C,C,\dots,C)$ *is Pareto optimal if and only if* $\sum_{j=1}^{n}\Delta p_j > c/b$*.*

In $(C,C,\dots,C)$, a player can only change strategy to D. If some players change their strategy to D, cooperators will suffer a decreased pay-off. However, if all players defect, it may be possible for all of them to enjoy an increased pay-off. The inequality $B_C(n) > B_D(0)$, which is equivalent to $p_n - p_0 > c/b$ or $\sum_{j=1}^{n}\Delta p_j > c/b$, assures that $(C,C,\dots,C)$ is Pareto optimal.

Result 6 *The strategy profile with* i *cooperators,* $i=0,1,\dots,n-1$*, is Pareto optimal if and only if* $\sum_{j=i+1}^{n}\Delta p_j < c/b$*.*

For a strategy with i ($i=0,1,\dots,n-1$) cooperators, all players experience an increased pay-off if some additional players cooperate. The inequality $B_D(i) > B_C(i+j)$, where j ($j=1,\dots,i+1$) represents additional cooperators, ensures that the strategy profile is Pareto optimal. $B_D(i) > B_C(i+j)$ is equivalent to $\Delta p_{i+j}+\ldots+\Delta p_{i+1} < c/b$ or $\sum_{k=i+1}^{j}\Delta p_k < c/b$. Since Δp_i is increasing, it is sufficient to require $\sum_{j=i+1}^{n}\Delta p_j < c/b$.

2. Size effect due to genetic drift

It is shown that the group size effect due to 'genetic drift' is negative for the weight-lifting model. We present this result here because it requires further assumptions that are out of scope of or not of direct relevance to the results in the main text, i.e., stochastic dynamics, the Moran process and weak selection.

Let us focus on one player in the total of n players. His/her pay-offs for taking A and B strategies are denoted as a_j and b_j, respectively, when $n-j$ players from among the other $n-1$ players take A (the rest $j-1$ players are B). Considering population dynamics of a Moran process with frequency-dependent fitness under the assumption of weak selection, Kurokawa and Ihara (2009) showed that the group size effect is positive or negative depending on the sign of

$$\alpha = \sum_{k=1}^{n} k(a_k - b_k).$$

More specifically, they obtained this result from the condition of whether $\rho_A > 1/N$ or $\rho_A < 1/N$, where ρ_A is the fixation probability of A, i.e., the probability that a population with a single player A reaches a population with all players playing A. Moreover, it is assumed that the n players are randomly selected from a larger population of size N. This is the assumption of an unstructured population.

It is straightforward to apply this result to the weightlifting game. A and B are regarded as C and D, respectively. Substituting $a_j = bp_{n-j+1} - c$ and $b_j = bp_{n-j}$, we have

$$\alpha = \sum_{k=1}^{n} k(bp_{n-k+1} - c - bp_{n-k}).$$

The middle term of c gives a negative contribution $-n(n+1)c/2$. The first and the last terms are collectively simplified by noting

$$\sum_{k=1}^{n} kp_{n-k} = \sum_{j=1}^{n} jp_{n-j+1} + (n+1)p_0 - 1.$$

Accordingly, we obtain

$$\alpha = b(1-(n+1)p_0) - \frac{n(n+1)}{2}c.$$

While the b term may not only stay positive but may grow large due to its n-dependence, eventually, α should become negative for a sufficiently large value of n. In other words, $\rho_C < 1/N$, and the group size effect is negative. However, it should be remembered that this simple (and seemingly trivial) result does not exclude a possibility to reach an all-C population through a non-trivial strategy like the tit-for-tat strategy for the iterated prisoner's dilemma.

Kurokawa, S. & Ihara, Y. 2009 Emergence of cooperation in public goods games. *Proc. R. Soc. B* **276**, 1379-1384. (doi: 10.1098/rspb.2008.1546)

Supplementary Figure

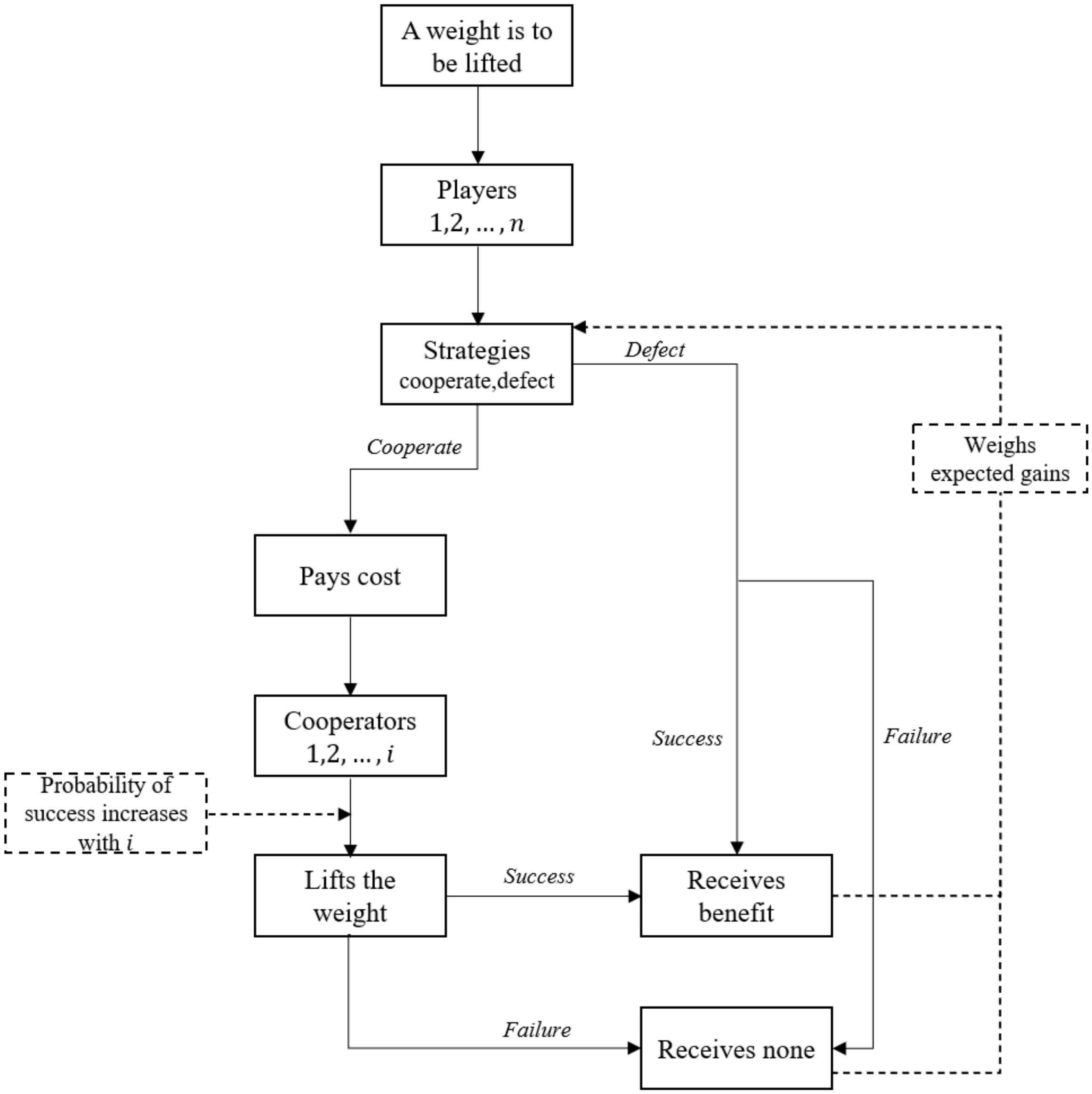

Figure S1: A flowchart of the *n*-player weightlifting game. In this game, players decide whether to cooperate or defect in carrying the weight. Cooperators need to pay a cost. The weightlifting can either succeed or fail. In case of success, all players receive a benefit. In case of failure, all players receive nothing. The player's pay-off depends on the benefit, cost and probability of success. Each player decides whether to cooperate or defect so as to maximize the expected gain.
